# Leadership, autonomy, and organizational trust as predictors of teacher wellbeing and job satisfaction: a cross-cultural study based on PISA 2022 data

**DOI:** 10.3389/fpsyg.2025.1703458

**Published:** 2026-01-02

**Authors:** Servet Üztemur, Ali Gökalp, Abdurrahman İlğan, Erkan Dinç

**Affiliations:** 1 Department of Turkish and Social Sciences Education, Faculty of Education, Anadolu University, Eskişehir, Türkiye; 2 Department of Educational Sciences, Gaziantep University, Gaziantep, Türkiye; 3 Department of Educational Sciences, Faculty of Education, Izmir Democracy University, Izmir, Türkiye; 4 Department of Primary Education, Faculty of Education, Anadolu University, Eskişehir, Türkiye

**Keywords:** cross-cultural comparison, job satisfaction, leadership, organizational trust, professional autonomy, teacher wellbeing

## Abstract

**Introduction:**

Leadership behaviors play a critical role in shaping educators’ psychological wellbeing and job satisfaction. However, less is known about the mechanisms through which leadership influences these outcomes across education systems with differing performance levels. This study examines the mediating roles of professional autonomy and organizational trust in the relationship between leadership behaviors, wellbeing, and job satisfaction among educators in different PISA-performing economies.

**Methods:**

Using PISA 2022 teacher data, this study analyzes responses from educators in three economies with contrasting PISA rankings: Brazil (low-performing; *N* = 4,051), Malaysia (medium-performing; *N* = 3,711), and Macao (high-performing; *N* = 1,656). Descriptive statistics and mediation analyses were employed to examine both direct and indirect effects of leadership behaviors on employee wellbeing and job satisfaction through professional autonomy and organizational trust.

**Results:**

The findings reveal that leadership behaviors are directly associated with both wellbeing and job satisfaction across all three economies. In addition, professional autonomy and organizational trust function as significant mediators in these relationships. The strength and configuration of the mediation pathways differ across the three economies, indicating context-specific patterns rather than a single uniform mediation model.

**Discussion:**

These results contribute to the organizational behavior and educational leadership literature by demonstrating that supportive leadership practices enhance educators’ psychological wellbeing and job satisfaction through increased autonomy and trust. Importantly, the varying magnitude of these effects across economies highlights the influence of systemic and contextual factors, suggesting that leadership interventions should be tailored to national and organizational contexts rather than applied uniformly across education systems.

## Introduction

Teachers’ wellbeing is essential for quality education. A fundamental condition for teachers to effectively meet professional demands is job satisfaction and low levels of burnout symptoms (Martinsone et al., 2024; Schulze-Hagenest et al., 2023). The high levels of stress inherent in the teaching profession, coupled with resulting low self-esteem, decreased job satisfaction, and an increased risk of burnout, create common problems that adversely affect teachers’ wellbeing (Acton and Glasgow, 2015; Hoque et al., 2023). Improved wellbeing enhances teachers’ job satisfaction and positive feelings toward their profession, encourages participation in professional development activities. Furthermore, teacher wellbeing is widely recognized as a critical factor for maintaining a sustainable and effective workforce in education (Hussain et al., 2022; McLean and Sandilos, 2022). Teacher wellbeing is a comprehensive and multifaceted concept encompassing physical, social, emotional, and psychological dimensions (Kern et al., 2014). The adverse effects of deficiencies in teachers’ competencies on their wellbeing are often highlighted (Falecki and Mann, 2021). This can result in diminished teacher performance and reduced teaching effectiveness, creating significant obstacles to maintaining overall educational quality. Accordingly, teacher wellbeing has become a central concern in international debates on educational quality and sustainability, particularly in the face of increasing workloads, staff shortages, and changing role expectations (Hussain et al., 2022; McLean and Sandilos, 2022).

School climate plays a crucial role in maintaining teacher wellbeing. Positive school climates decrease teacher absenteeism and enhance their sense of belonging (Hoy and Miskel, 2012). Moreover, collaborative and supportive environments enhance teachers’ job satisfaction, improve their performance, and positively impact learning outcomes (Reeves et al., 2017). The leadership behaviors exhibited by school administrators are also important factors affecting teachers’ wellbeing (Hoy and Miskel, 2012; Van der Vyver et al., 2020). While empowering leadership approaches positively impact teachers’ wellbeing, they also foster a more inclusive working environment by reducing perceptions of organizational exclusion (Okçu et al., 2025). Consequently, prioritizing teachers’ wellbeing is crucial for their personal and professional lives and for enhancing students’ learning experiences. Teachers who feel satisfied at work are less vulnerable to stress and burnout (Schulze-Hagenest et al., 2023). As a result, teachers have a more positive self-view, which boosts students’ motivation to learn and ultimately improves the quality of teaching (Klusmann et al., 2021; Skaalvik and Skaalvik, 2020). Understanding the factors affecting teacher wellbeing is crucial for teachers’ personal and professional lives and for students’ learning experiences. However, much of the existing research has tended to focus on specific antecedents of teacher wellbeing in single-country settings, with less attention to how different organizational resources such as leadership, autonomy, trust, and job satisfaction operate together across diverse education systems (Mgaiwa and Hamis, 2022; Toropova et al., 2021). Large-scale international datasets such as PISA provide a unique opportunity to address this gap by modeling these relationships comparably across contrasting educational contexts (OECD, 2014).

### The relationship between educational leadership, teachers’ wellbeing, and job satisfaction

Principals are a crucial component in maintaining a positive school climate and fostering supportive professional relationships within the school environment (Dicke et al., 2022; Skaalvik, 2023). To reduce teachers’ work-related stress, school administrators need to plan, adapt, and organize their work tasks accordingly and provide adequate support (Martinsone et al., 2024). The impact of principals’ leadership characteristics on teachers has been the focus of various studies (Qi et al., 2025; Bellibaş et al., 2020). Research has shown that principals’ leadership characteristics and practices significantly affect teachers’ wellbeing and professional practices. For instance, teachers’ subjective wellbeing (Qi et al., 2025), job satisfaction (Bellibaş et al., 2020; Qi et al., 2025), work engagement, and emotional exhaustion (Zhong et al., 2020) can all be influenced by principal leadership. Studies indicate that different leadership styles can have various impacts on teachers’ professional performance (Abu Nasra and Arar, 2020). Instructional leadership involves practices such as principals observing classroom teaching, fostering collaboration among teachers, and ensuring that teachers take responsibility for enhancing their professional skills (Bellibaş et al., 2020; Hallinger, 2005; Gumus et al., 2018). This leadership style not only positively impacts teachers’ teaching quality but also enhances teacher collaboration and job satisfaction (Bellibaş et al., 2020). Another key leadership approach, distributed leadership, focuses on sharing leadership roles among teaching staff and encouraging teachers’ active participation in school-level decision-making processes (Bellibaş et al., 2020; Harris, 2008; Liu et al., 2018). In a distributed leadership model, when principals engage teachers in decision-making processes, teachers are more likely to collaborate and experience greater job satisfaction (Bellibaş et al., 2020).

In a study conducted across various organizational contexts (Zhong et al., 2020), leadership traits such as leader humility were positively associated with employee wellbeing and negatively associated with emotional exhaustion. Studies indicate that a substantial relationship exists between educational leadership and teachers’ job satisfaction (Crisci et al., 2019; Nguni et al., 2006; Halim et al., 2021). School principals play a crucial role in attracting, retaining, motivating, and professionally developing teachers; job satisfaction among teachers is vital for enhancing their performance, increasing retention, strengthening their commitment to the institution, and reducing job stress (Halim et al., 2021; Mgaiwa and Hamis, 2022; Toropova et al., 2021). Therefore, the importance of educational leadership among the factors affecting teacher satisfaction is undeniable, and deficiencies in educational leadership or negative leadership styles can lead teachers to experience job dissatisfaction and even intention to leave the profession (Al-Mahdy et al., 2016; Saadaoui et al., 2024).

It is emphasized that the effects of school administrators with different leadership styles on teacher satisfaction may vary, and that leadership style is a crucial factor in teachers’ wellbeing and commitment to the school (Heidmets and Liik, 2014; Mgaiwa and Hamis, 2022). Specifically, transformational leadership, which involves motivating and inspiring teachers while caring for their individual development, is generally positively and significantly associated with teachers’ job satisfaction (Heidmets and Liik, 2014; Nguni et al., 2006; Basar et al., 2021). Factors such as individual attention and inspirational motivation have been reported as predictors of transformational leadership, which enhances teachers’ job satisfaction (Basar et al., 2021). Furthermore, a meta-analysis examining the relationship between principal leadership and teacher job satisfaction (Juhji et al., 2022) found that principals’ transformational leadership significantly impacts teachers’ job satisfaction. Servant leadership, which empathizes with teachers and prioritizes their needs and wellbeing (Qiu and Dooley, 2019), similarly has a positive impact on job satisfaction, and teachers who work with principals exhibiting servant leadership qualities are more satisfied with their jobs (Von Fischer and De Jong, 2017; Saadaoui et al., 2024). A significant and positive relationship exists between transactional leadership and job satisfaction (Halim et al., 2021). Laissez-faire leadership has also been shown to be significantly and positively associated with job satisfaction (Rexha and Buleshkaj, 2024; Shaari et al., 2022). In contrast, passive-avoidant and autocratic leadership styles have been reported to negatively impact teachers’ job satisfaction (Halim et al., 2021; Rexha and Buleshkaj, 2024). A meta-analysis (Cogaltay et al., 2016) examining the influence of educational leadership on teachers’ job satisfaction found that educational leadership generally has a strong, positive effect on job satisfaction. Specifically, transformational, cultural, and instructional leadership styles show strong positive correlations with job satisfaction. The studies cited above indicate that the leadership behaviors and styles displayed by school principals significantly influence aspects of teachers’ wellbeing and professional lives, including job satisfaction, emotional state, and collaboration. It would not be wrong to say that educational leadership stands out as a significant factor in motivating teachers and enhancing their performance, as well as in shaping the overall school climate. From this perspective, principal leadership can be conceptualized as an organizational resource that has the potential to foster teachers’ wellbeing both directly and indirectly through the working conditions it creates (Hoy and Miskel, 2012; Martinsone et al., 2024).

### The mediating role of teacher autonomy and organizational trust

Teacher autonomy refers to teachers’ decision-making authority and responsibility for matters related to the school, as well as for planning and implementing teaching activities (Yurtseven and Hoşgörür, 2021). It is emphasized that teachers’ professional autonomy is crucial for enhancing job satisfaction, productivity, and wellbeing; however, teacher autonomy is a relational and contextual structure, with educational leadership being one of its primary dimensions (Keddie et al., 2024). When teachers perceive a trust-based relationship at school, they are more likely to demonstrate leadership behaviors (Kılınç et al., 2021). A systematic review (Cansoy and Polatcan, 2019) examining the relationship between principals’ leadership behaviors and teachers’ organizational commitment emphasized that creating a work culture based on justice, respect, and trust is one leadership behavior that enhances teachers’ organizational commitment. This study demonstrated that humanistic leadership styles of principals, such as transformational and servant leadership, positively impact teachers’ school engagement. Okçu et al. (2025) found that empowering leadership positively affected teachers’ wellbeing at work and significantly reduced their perceptions of organizational exclusion. Trust in the institution and the administrator is an important factor related to teachers’ job satisfaction and organizational commitment (Karataş and Güleş, 2010). Another concept closely related to job satisfaction is teacher autonomy. A 2020 report by the National Foundation for Educational Research highlights that low autonomy, particularly over professional development goals, negatively impacts teachers’ job satisfaction and their intention to remain in the profession. Therefore, school leaders should support teacher autonomy (Worth and den Brande, 2020).

Empirical studies have consistently shown a significant positive relationship between teachers’ autonomy behaviors and job satisfaction (Alkan et al., 2025; Dilekçi, 2022; Jerrim et al., 2023; Peng et al., 2022). In addition to job satisfaction, teacher autonomy positively affects teacher engagement and negatively affects emotional exhaustion (Skaalvik and Skaalvik, 2014). This suggests that teachers who feel autonomous in the work environment may be more satisfied with their jobs. On the other hand, situations such as principals not providing teachers with autonomous working spaces in the school environment, poor cooperation, and a lack of teachers’ participation in decision-making are considered factors that reduce teachers’ commitment to the organization (Choi and Tang, 2009). Taken together, these findings indicate that autonomy and organizational trust are central mechanisms through which leadership practices are translated into teachers’ experiences of their work. Nevertheless, relatively few studies have simultaneously tested teacher autonomy and organizational trust as parallel and serial mediators between educational leadership and wellbeing outcomes, and even fewer have explored whether these mechanisms operate similarly across different economies using comparable large-scale data (Cansoy and Polatcan, 2019; Peng et al., 2022). Investigating these mediating pathways in multiple contexts can therefore clarify whether similar leadership practices are associated with teacher wellbeing through common mechanisms or whether the pattern of associations varies across education systems.

### Purpose of the study

The purpose of this study is to examine the relationships between teachers’ professional autonomy, job satisfaction, perceptions of organizational trust, wellbeing, and perceptions of educational leadership in three different economies (Brazil, Malaysia, and Macao), which are categorized differently in terms of PISA performance rankings and are geographically distant from each other. By focusing on Brazil, Malaysia, and Macao, which differ in their geographic locations, socioeconomic conditions, and PISA performance, the study seeks to identify both common and context-specific patterns in how leadership, autonomy, organizational trust, and job satisfaction relate to teacher wellbeing (OECD, 2014; Sahlberg, 2013). In doing so, this research extends prior work by modeling multiple mediators simultaneously and by applying the same analytical framework to large nationally representative teacher samples from three contrasting education systems. In line with these aims, the following research questions (RQs) were addressed, and hypotheses were tested separately for each economy.

*RQ1:* What is the level of teachers' professional autonomy, job satisfaction, organizational trust, well-being, and perceptions of educational leadership? Does this level differ significantly according to economies?

*H1:* Organizational trust mediates the relationship between educational leadership and well-being.

*H2:* Autonomy mediates the relationship between educational leadership and well-being.

*H3:* Job satisfaction mediates the relationship between educational leadership and well-being.

*H4:* The relationship between educational leadership and well-being is serially mediated by organizational trust and job satisfaction.

*H5:* The relationship between educational leadership and well-being is serially mediated by autonomy and job satisfaction.

**Figure 1:**
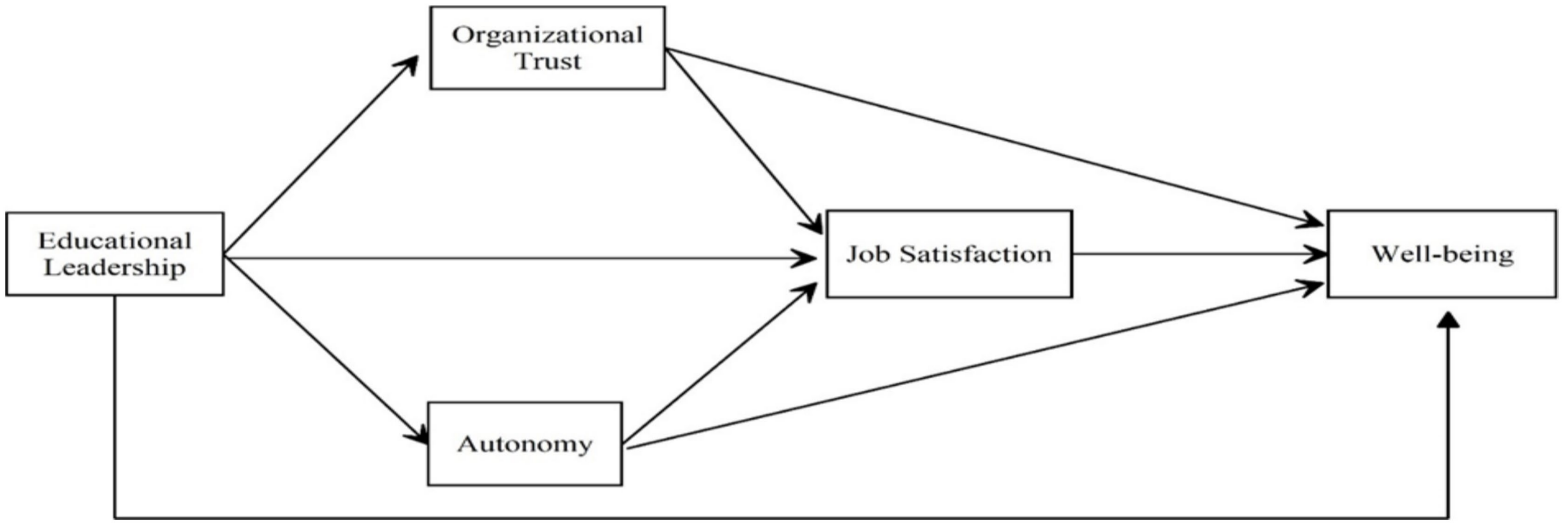
The hypothetical model.

Based on the conceptual framework outlined above and the literature, a model (Figure 1) was proposed, and the hypotheses above were tested in line with this model.

Based on the conceptual framework outlined above and the literature, a model (Figure 1) was proposed, and the hypotheses above were tested in line with this model.

## Method

### The population and sample

The data source for this study is the PISA 2022 teacher data set, which is publicly available on the OECD website.^1^ In PISA 2022, the Teacher Questionnaire (TQ) was offered as an optional international option and was implemented by a limited number of countries/economies, including Brazil, Macao (China), and Malaysia. The survey aimed to understand students’ learning environments and teachers’ practices; the data obtained was intended to be used alongside student data to describe the learning environment of typical 15-year-old students in that country. Sampling was conducted using simple random sampling from teacher lists. Teachers were selected either from the entire list of teachers or from a cluster target size (typically 10 for mathematics teachers and 15 for other teachers), determined by the population size at that school. The selection of teachers was limited to those teaching the modal classes attended by 15-year-old students at the school, to ensure alignment with the survey’s objectives. To ensure adequate representation in the teacher sample, weighting procedures were applied, as in the student sample. The purpose of weighting was to calculate teacher weights to ensure that the data obtained from the teacher survey represented the correct number of teachers within the population in the analysis. Teacher weights were calculated based on school weights and intra-school teacher weights (the inverse of selection probability), and adjustments were made according to school/student non-response. This ensured the representation of the total populations of mathematics and non-mathematics teachers (OECD, 2024).

Three of the 18 countries/economies were selected from the dataset. Accordingly, Macao, which has the highest PISA performance among the countries with available teacher data, Malaysia, which has moderate performance, and Brazil, which is below average and on a different continent, were included in the analysis, while the others were excluded.

In Macao and Brazil, female teachers outnumbered their male counterparts by more than half, while in Malaysia, female teachers made up three-quarters of the sample. The mean age of the teachers was, in descending order, Brazil (mean = 42.44), Malaysia (mean = 42.22), and Macao (mean = 38.25). The average age of Brazilian and Malaysian teachers is quite similar, whereas Macao teachers have a younger population than teachers in other countries. Teachers’ professional seniority ranges from more to less, Malaysia (mean = 16.74), Brazil (mean = 15.66), and Macao (mean = 13.58); and the length of service in the same school ranges from more to less, Macao (mean = 10.67), Malaysia (mean = 9.91), and Brazil (mean = 7.53).

### Measures

In this study, five different scales were utilized: Principal Leadership Scale (PLS), Teacher Autonomy Scale (TAS), Job Satisfaction Scale (JSS), Organizational Trust Scale (OTS), and WellBeing of Teachers (WBT). The measurement invariance of these scales across the three economies (Brazil, Macao, and Malaysia) was examined using Multi-Group Confirmatory Factor Analysis (MG-CFA) with the Robust Maximum Likelihood (ML) estimator to account for the ordinal nature of the data. In the invariance testing process, first, a configural model was established to verify that the factor structure was consistent across groups. Subsequently, metric invariance was tested by constraining factor loadings to be equal across the three economies. Since the primary objective of this study is to examine structural relationships and mediation effects rather than comparing latent means, establishing metric invariance was considered sufficient for the validity of the proposed model (Cheung and Rensvold, 2002). Model fit was evaluated using widely accepted indices. Accordingly, Comparative Fit Index (CFI) and Tucker-Lewis Index (TLI) values of 0.90 and above were considered indicative of acceptable fit (Bentler, 1990). For the Root Mean Square Error of Approximation (RMSEA) and Standardized Root Mean Square Residual (SRMR), values below 0.08 are considered a good fit, while values up to 0.10 are regarded as an acceptable fit (Browne and Cudeck, 1992; Hu and Bentler, 1999). In addition to the measurement invariance analysis, the internal consistency coefficients for the scales were calculated using Cronbach’s Alpha. An Alpha value of 0.70 or higher is generally considered to indicate sufficient reliability (George and Mallery, 2019).

#### Principal Leadership Scale (PLS)

The Principal Leadership Scale (PLS), derived from the PISA 2022 dataset (coded as TC_253), measures teachers’ perceptions of school principals’ leadership behaviors using seven items. Responses were rated on a 4-point Likert scale. The unidimensional structure of the scale was tested across the three economies using MG-CFA with the robust ML estimator. The results confirmed that the configural model fit the data well in all groups (*X*^2^_(42)_ = 397.07, CFI = 0.992, TLI = 0.988, RMSEA = 0.051, SRMR = 0.051). Furthermore, the metric invariance test, which constrains factor loadings to be equal across groups, demonstrated a negligible decrease in model fit (ΔCFI = 0.003), supporting the measurement invariance of the scale. The internal consistency of the scale was high across all economies, with Cronbach’s Alpha coefficients of 0.92 for Brazil, 0.90 for Macao, and 0.94 for Malaysia.

#### Teacher Autonomy Scale (TAS)

The Teacher Autonomy Scale (TAS) assesses the level of professional autonomy perceived by teachers using items derived from the PISA 2022 questionnaire (coded as TC_246). The original scale consisted of seven items rated on a 4-point Likert scale. During the preliminary analysis, one item (item 6) was removed to ensure structural consistency across groups due to low factor loadings in the Malaysian sample. The MG-CFA results for the remaining six items indicated an excellent model fit for the configural model (*X*^2^_(27)_ = 92.07, CFI = 0.994, TLI = 0.989, RMSEA = 0.027, SRMR = 0.033). Subsequently, metric invariance was established by constraining factor loadings to be equal across the three economies. The resulting change in the CFI (ΔCFI = 0.008) was within the recommended threshold of 0.010, confirming that the measurement structure is invariant across Brazil, Macao, and Malaysia. The scale demonstrated satisfactory internal consistency, with Cronbach’s Alpha coefficients of 0.82 for Brazil, 0.81 for Macao, and 0.80 for Malaysia.

#### Job Satisfaction Scale (JSS)

Teachers’ job satisfaction was measured using the Job Satisfaction Scale (JSS) derived from the PISA 2022 Teacher Questionnaire (coded as TC_198). The scale consists of items rated on a 4-point Likert scale ranging from “strongly disagree” to “strongly agree,” where higher scores indicate greater job satisfaction. Psychometric evaluations and Multi-Group CFA results confirmed that the scale’s unidimensional structure was valid and strictly invariant across the three economies. The measurement model demonstrated excellent fit indices under the configural model (*X*^2^_(9)_ = 16.79, CFI = 0.998, TLI = 0.994, RMSEA = 0.016, SRMR = 0.019), and metric invariance was fully supported with a negligible change in fit indices (ΔCFI < 0.01). The internal consistency coefficients (Cronbach’s Alpha) for the scale were calculated as 0.79 for Brazil, 0.77 for Macao, and 0.80 for Malaysia, indicating reliability across all groups.

#### Organizational Trust Scale (OTS)

The Organizational Trust Scale (OTS) measures teachers’ level of trust in their colleagues and school administration. The scale consists of five items derived from the PISA 2022 questionnaire (coded as TC_241). MG-CFA with the robust ML estimator was conducted to test the measurement invariance across Brazil, Macao, and Malaysia. The configural model demonstrated a good fit to the data (*X*^2^_(15)_ = 140.18, CFI = 0.976, TLI = 0.953, RMSEA = 0.051, SRMR = 0.071), indicating a consistent factor structure across groups. Following this, metric invariance was tested by constraining factor loadings to be equal across the three economies. The model fit remained excellent, and the change in the CFI (ΔCFI = 0.001) was negligible and well within the acceptable threshold, supporting full metric invariance. The scale showed high internal consistency, with Cronbach’s Alpha coefficients of 0.88 for Brazil, 0.81 for Macao, and 0.87 for Malaysia.

#### Wellbeing of teachers (WBT)

Teacher wellbeing was assessed using the items derived from the PISA 2022 questionnaire (coded as TC_237). The scale originally consisted of six items measuring teachers’ general wellbeing. The items were treated as a single, unidimensional construct representing teachers’ general wellbeing. During the preliminary analysis, one item (TC237Q06JA) was removed due to low factor loading to ensure construct validity and measurement stability across groups. The MG-CFA with the robust ML estimator was conducted on the remaining five items. The results indicated an exceptional fit for the configural model (*X*^2^_(15)_ = 23.88, CFI = 0.999, TLI = 0.997, RMSEA = 0.013, SRMR = 0.024). Subsequently, metric invariance was tested by constraining factor loadings to be equal across the three economies. The model retained excellent fit indices, and the change in the CFI (ΔCFI = 0.001) was negligible, confirming the invariance of the measurement structure across Brazil, Macao, and Malaysia. The internal consistency of the scale was high, with Cronbach’s Alpha coefficients of 0.83 for Brazil, 0.86 for Macao, and 0.84 for Malaysia.

### Statistical analysis

#### Preliminary analysis

Prior to the primary analyses, the assumption of normality was verified, as this is a prerequisite for the reliability of significance tests and the construction of confidence intervals (Field, 2018). In the preliminary analyses, descriptive statistics, Pearson product–moment correlation analyses, and one-way ANOVA with Tukey HSD adjustment were applied. Consistent with Field's (2018) recommendation to better estimate population parameters and interpret effect precision, 95% confidence intervals (CIs) were calculated and reported alongside the correlation coefficients. Furthermore, eta-squared effect sizes (*η2*) were calculated for statistically significant differences detected in the one-way ANOVA. According to Cohen (1988), the threshold values for eta-squared effect sizes are classified as small (*η2* = 0.01), medium (*η2* = 0.06), and large (*η2* = 0.14). Jeffreys’s Amazing Statistics Program (JASP) version 0.19.2 was used for CFA, while IBM SPSS version 20 was utilized for all other analyses. Statistical significance was defined as a *p*-value < 0.05.

#### Serial mediation analyses

SPSS PROCESS macro Model 80, developed by Hayes (2022), was used in serial mediation analyses. In the model, the path shown in Figure 1, with wellbeing as the dependent variable and principal leadership as the independent variable, was tested. Then, the models in which organizational trust, autonomy, and job satisfaction were dependent variables were tested. In the next stage, the serial mediation effect of organizational trust and job satisfaction was tested in the model, with wellbeing as the dependent variable and principal leadership as the independent variable. In the final stage, the serial mediation effect of autonomy and job satisfaction was tested in the model, with wellbeing as the dependent variable and principal leadership as the independent variable. To determine whether the indirect effect coefficient is significant, it is tested using 5,000 bootstrap samples with a 95% confidence interval, as suggested by Hayes (2022). Significant effects are supported when the CI for the difference between the lower (LL) and upper (UL) limits does not contain zero (Hayes, 2022).

## Results

### Preliminary analysis

Table 1 presents descriptive statistics and ANOVA results for teachers’ wellbeing, job satisfaction, organizational trust, professional autonomy, and perceptions of school principal leadership by economy (i.e., Brazil, Macao, and Malaysia).

**Table 1:** Descriptive statistics and ANOVA results for variables examined by economy.

Variables	Brazil M (SD)	Macao M (SD)	Malaysia M (SD)	df	*F*	*p*	*η* ^2^
Teachers’ wellbeing	29.86 (4.91)^a^	28.43 (5.04)^a^	31.19 (4.86)^b^	2, 9,532	194.10	<0.001	0.039
Job satisfaction	24.88 (4.26)^b^	24.54 (3.75)^a^	26.42 (3.45)^c^	2, 9,611	209.30	<0.001	0.042
Organizational trust	16.22 (2.58)^a^	47.31 (14.91)^b^	46.69 (19.55)^b^	2, 9,802	6101.00	<0.001	0.555
Professional autonomy	21.21 (3.70)^c^	14.98 (2.32)^a^	16.30 (2.21)^b^	2, 9,802	3983.00	<0.001	0.448
Principal’s leadership	18.34 (5.40)^b^	17.92 (3.64)^a^	20.29 (3.68)^c^	2, 9,802	247.60	<0.001	0.048

df, degrees of freedom. Means with different superscripts (i.e., a, b, c) on the same row are significantly different from each other at the 0.05 level according to the Tukey HSD test. M, mean; SD, standard deviation.

As shown in Table 1, teachers’ wellbeing (*F*(2, 9,532) = 194.10, *p* < 0.001), job satisfaction (*F*(2, 9,611) = 209.30, *p* < 0.001), and organizational trust (*F*(2, 9,802) = 6101.00, *p* < 0.001), professional autonomy (*F*(2, 9,802) = 3983.00, *p* < 0.001), and perceptions of school principal leadership (*F*(2, 9,802) = 247.60, *p* < 0.001); differed statistically significantly according to the economy in which the teachers were located. When examining the effect sizes of the between-group differences, a large effect was observed in the variables of organizational trust (*η2* = 0.555) and professional autonomy (*η2* = 0.448); while the difference was found to be small in the variables of wellbeing (*η2* = 0.039), job satisfaction (*η2* = 0.042), and school principal leadership (*η*2 = 0.048).

According to the Tukey HSD *post-hoc* test results, teachers’ wellbeing scores in Malaysia (M = 31.19) are significantly higher than those in Brazil (M = 29.86) and Macao (M = 28.43). No significant difference was found between Brazil and Macao. In terms of job satisfaction, the three economies differed significantly. The highest job satisfaction was observed in Malaysia (M = 26.42), while the lowest was in Macao (M = 24.54). The level of organizational trust among teachers in Brazil (M = 16.22) is lower than in the other two countries and significantly different. There is no significant difference between Macao (M = 47.31) and Malaysia (M = 46.69). In terms of professional autonomy, Brazil (M = 21.21) has the highest average, followed by Malaysia (M = 16.30) and Macao (M = 14.98); the differences between all groups are significant. The three economies also differ statistically significantly in their perceptions of school principal leadership. The highest perceived leadership is among teachers in Malaysia (M = 20.29), followed by Brazil (M = 18.34) and Macao (M = 17.92). Table 2 presents correlations and descriptive statistics for the relevant variables.

**Table 2:** Correlations and descriptive statistics for the relevant variables.

Country	Variable	Skew	Kurt	(1)	(2)	(3)	(4)	(5)
Macao	1. Principal’s leadership	0.36	0.12	–	0.41 [0.37, 0.45]	0.16 [0.11, 0.20]	0.30 [0.25, 0.34]	0.26 [0.21, 0.30]
	2. Organizational trust	−0.23	1.74		–	0.31 [0.27, 0.35]	0.52 [0.49, 0.56]	0.45 [0.41, 0.49]
	3. Professional autonomy	0.03	0.04			–	0.32 [0.28, 0.36]	0.28 [0.24, 0.32]
	4. Job satisfaction	−0.13	0.29				–	0.55 [0.52, 0.59]
	5. Teachers’ wellbeing	−0.17	0.31					–
Malaysia	1. Principal’s leadership	−0.21	0.09	–	0.44 [0.42, 0.47]	0.27 [0.24, 0.30]	0.36 [0.33, 0.39]	0.27 [0.24, 0.30]
	2. Organizational trust	0.31	−0.15		–	0.32 [0.29, 0.35]	0.50 [0.48, 0.53]	0.45 [0.42, 0.47]
	3. Professional autonomy	−0.13	−0.10			–	0.28 [0.25, 0.31]	0.26 [0.23, 0.29]
	4. Job satisfaction	−0.22	−0.23				–	0.50 [0.48, 0.53]
	5. Teachers’ wellbeing	−0.15	−0.10					–
Brazil	1. Principal’s leadership	−0.02	−0.62	–	0.52 [0.50, 0.54]	0.28 [0.26, 0.31]	0.27 [0.25, 0.30]	0.20 [0.17, 0.23]
	2. Organizational trust	−0.36	0.56		–	0.33 [0.30, 0.35]	0.47 [0.44, 0.49]	0.35 [0.32, 0.37]
	3. Professional autonomy	−0.06	−0.28			–	0.28 [0.25, 0.31]	0.30 [0.28, 0.33]
	4. Job satisfaction	−0.38	−0.01				–	0.48 [0.45, 0.50]
	5. Teachers’ wellbeing	−0.31	0.37					–

Values in brackets indicate the 95% confidence interval (lower, upper level) for each correlation. Skew, skewness; Kurt, kurtosis. All correlations *p* < 0.001.

As shown in Table 2, the data distribution was assumed to be normal, as skewness and kurtosis values ranged within ±2.0 (George and Mallery, 2019). The analysis demonstrated statistically significant, positive correlations between all variables across the three economies (*p* < 0.001). The 95% confidence intervals for these coefficients are narrow, suggesting reliable estimates of the population parameters (Field, 2018).

### Serial mediation analyses results

Table 3 presents the beta coefficients (*b*) for the effect of principal leadership on wellbeing for each economy separately. Overall, the serial mediation model explained a significant proportion of variance in teachers’ wellbeing across the three economies (*R^2^* = 0.35 for Macao, *R^2^* = 0.31 for Malaysia, and *R^2^* = 0.27 for Brazil).

**Table 3:** Serial mediation effect of principal leadership on wellbeing.

Economy/path	*b*	SE	95% CI [LL, UL]
Macao (*N* = 1,656)			
Direct effects			
Principal leadership → organizational trust	0.41	0.01	[0.19, 0.24]
Principal leadership → autonomy	0.15	0.01	[0.09, 0.16]
Principal leadership → job satisfaction	0.09	0.01	[0.04, 0.11]
Principal leadership → wellbeing	0.05	0.02	[0.00, 0.10]
Organizational trust → job satisfaction	0.43	0.03	[0.62, 0.77]
Autonomy → job satisfaction	0.17	0.02	[0.13, 0.22]
Organizational TRUST → wellbeing	0.19	0.05	[0.30, 0.51]
Autonomy → wellbeing	0.08	0.02	[0.06, 0.17]
Job satisfaction → wellbeing	0.41	0.03	[0.49, 0.62]
Indirect effects			
Principal leadership → organizational trust → wellbeing	0.21	0.01	[0.18, 0.25]
Principal leadership → autonomy → wellbeing	0.08	0.01	[0.05, 0.10]
Principal leadership → job satisfaction → wellbeing	0.01	0.01	[0.01, 0.02]
Principal leadership → organizational trust → job satisfaction → wellbeing	0.07	0.01	[0.06, 0.09]
Principal leadership → autonomy → job satisfaction → wellbeing	0.01	0.01	[0.01, 0.02]
Total indirect effect	0.21	0.01	[0.18, 0.25]
Malaysia (*N* = 3,711)			
Direct effects			
Principal leadership → organizational trust	0.44	0.01	[0.21, 0.24]
Principal leadership → autonomy	0.27	0.01	[0.20, 0.25]
Principal leadership → job satisfaction	0.16	0.01	[0.10, 0.15]
Principal leadership → wellbeing	0.01	0.01	[−0.02, 0.04]
Organizational trust → job satisfaction	0.40	0.02	[0.58, 0.67]
Autonomy → job satisfaction	0.11	0.01	[0.08, 0.13]
Organizational trust → wellbeing	0.24	0.03	[0.46, 0.60]
Autonomy → wellbeing	0.08	0.01	[0.07, 0.14]
Job satisfaction → wellbeing	0.36	0.02	[0.46, 0.55]
Indirect effects			
Principal leadership → organizational trust → wellbeing	0.11	0.01	[0.09, 0.12]
Principal leadership → autonomy → wellbeing	0.02	0.01	[0.01, 0.03]
Principal leadership → job satisfaction → wellbeing	0.06	0.01	[0.04, 0.07]
Principal leadership → organizational trust → job satisfaction → wellbeing	0.06	0.01	[0.05, 0.07]
Principal leadership → autonomy → job satisfaction → wellbeing	0.01	0.01	[0.01, 0.01]
Total indirect effect	0.26	0.01	[0.24, 0.28]
Brazil (*N* = 4,051)			
Direct effects			
Principal leadership → organizational trust	0.52	0.01	[0.24, 0.26]
Principal leadership → autonomy	0.29	0.01	[0.18, 0.22]
Principal leadership → job satisfaction	0.02	0.01	[−0.01, 0.04]
Principal leadership → wellbeing	0.01	0.01	[−0.04, 0.02]
Organizational trust → job satisfaction	0.41	0.02	[0.61, 0.72]
Autonomy → job satisfaction	0.14	0.01	[0.13, 0.19]
Organizational trust → wellbeing	0.12	0.03	[0.17, 0.30]
Autonomy → wellbeing	0.16	0.01	[0.17, 0.24]
Job satisfaction → wellbeing	0.38	0.01	[0.40, 0.47]
Indirect effects			
Principal leadership → organizational trust → wellbeing	0.07	0.01	[0.05, 0.08]
Principal leadership → autonomy → wellbeing	0.05	0.01	[0.04, 0.06]
Principal leadership → job satisfaction → wellbeing	0.01	0.01	[0.00, 0.02]
Principal leadership → organizational trust → job satisfaction → wellbeing	0.08	0.01	[0.07, 0.09]
Principal leadership → autonomy → job satisfaction → wellbeing	0.02	0.01	[0.01, 0.02]
Total indirect effect	0.22	0.01	[0.19, 0.24]

b, coefficients; SE, standard error; CI, confidence interval; LL, lower limit; UL, upper limit. Bootstrap sample size = 5,000. All coefficients are significant if the 95% CI does not contain zero.

Table 3 shows that the mediating effect of organizational trust in the relationship between principal leadership and wellbeing is statistically significant in all three economies (Macao: *b* = 0.21, *SE* = 0.01; Malaysia: *b* = 0.11, *SE* = 0.01; Brazil: *b* = 0.07, *SE* = 0.01). There were no zero values between the confidence intervals of the interaction value in each of the three economies (Macao: 95% CI [0.18, 0.25]; Malaysia: 95% CI [0.09, 0.12]; Brazil: 95% CI [0.05, 0.08]). Accordingly, H1 is confirmed for all three economies.

The mediating effect of autonomy on the relationship between principal leadership and wellbeing was statistically significant in all three economies (Macao: *b* = 0.08, *SE* = 0.01; Malaysia: *b* = 0.02, *SE* = 0.01; Brazil: *b* = 0.05, *SE* = 0.01). There were no zero values between the confidence intervals of the interaction value in each of the three economies (Macao: 95% CI [0.05, 0.10]; Malaysia: 95% CI [0.01, 0.03]; Brazil: 95% CI [0.04, 0.06]). Accordingly, H2 is confirmed for all three economies.

The mediating effect of job satisfaction on the relationship between principal leadership and wellbeing was statistically significant in Macao and Malaysia (Macao: *b* = 0.01, *SE* = 0.01; Malaysia: *b* = 0.06, *SE* = 0.01). There were no zero values between the confidence intervals of the interaction value in Macao (95% CI [0.01, 0.02]) and Malaysia (95% CI [0.04, 0.07]). Accordingly, H3 is confirmed in Macao and Malaysia, but not in Brazil where the confidence interval included zero.

The serial mediation effect of organizational trust and job satisfaction in the relationship between principal leadership and wellbeing is statistically significant in all three economies (Macao: *b* = 0.07, *SE* = 0.01; Malaysia: *b* = 0.06, *SE* = 0.01; Brazil: *b* = 0.08, *SE* = 0.01). There were no zero values between the confidence intervals of the interaction value in each of the three economies (Macao: 95% CI [0.06, 0.09]; Malaysia: 95% CI [0.05, 0.07]; Brazil: 95% CI [0.07, 0.09]). Accordingly, H4 is confirmed for all three economies.

The serial mediation effect of autonomy and job satisfaction in the relationship between principal leadership and wellbeing was statistically significant in all three economies (Macao: *b* = 0.01, *SE* = 0.01; Malaysia: *b* = 0.01, *SE* = 0.01; Brazil: *b* = 0.02, *SE* = 0.01). The significance of the indirect effect was tested with 5,000 bootstrapping samples at a 95% confidence interval. There were no zero values between the confidence intervals of the interaction value in each of the three economies (Macao: 95% CI [0.01, 0.02]; Malaysia: 95% CI [0.01, 0.01]; Brazil: 95% CI [0.01, 0.02]). Accordingly, H5 is confirmed for all three economies.

## Discussion

This study examined the patterns of relationships between teachers’ perceptions of principal leadership, wellbeing, professional autonomy, job satisfaction, and organizational trust in three PISA 2022 economies, Macao, Malaysia, and Brazil. In addition, the general tendencies of teachers in these three education systems regarding the variables analyzed were revealed. Rather than treating these differences as fixed cultural traits, we interpret them as economy-level patterns that reflect the interaction between leadership practices, working conditions, and broader system-level policies. The results obtained from descriptive statistics and mediation analyses, including correlation findings, are discussed under two separate headings, and various implications are drawn.

### Implications of descriptive statistics

Teachers’ professional autonomy levels varied significantly across the investigated economies, with Brazilian teachers reporting the highest autonomy, followed by their colleagues in Malaysia and Macao. The observation that Macao, a high-performing system in PISA, exhibits lower teacher autonomy compared to Brazil and Malaysia presents an interesting paradox. This pattern resonates with Sahlberg’s (2013) argument that high student achievement does not necessarily correlate with high levels of teacher autonomy in all contexts. The relationship between autonomy and achievement appears complex; as noted by Ayral et al. (2014), the relationship between autonomy and outcomes may depend heavily on the specific teaching domain and the broader school context. Consequently, these differences should be viewed as descriptive configurations of each education system rather than direct indicators of cultural superiority.

Regarding job satisfaction, Malaysian teachers reported significantly more positive views compared to their counterparts in Brazil and Macao. Given that job satisfaction is a critical predictor of teacher retention, performance, and reduced stress (Halim et al., 2021; Mgaiwa and Hamis, 2022; Toropova et al., 2021), the observed variations imply distinct professional experiences within these systems. While satisfaction levels were generally moderate to high, the distinct advantage observed in the Malaysian sample suggests a relatively more favorable perception of working conditions in that specific context.

A distinct disparity emerged in the domain of organizational trust. Unlike the high and statistically similar trust levels observed in the Asian economies of Macao and Malaysia, teachers in Brazil reported significantly lower levels of trust. Trust in colleagues and administration is fundamental for fostering organizational commitment (Karataş and Güleş, 2010), and the notably lower scores in Brazil point to potential systemic challenges in the relational fabric of schools. This contrasts sharply with the high-trust environments reported in the other two economies, highlighting an area that may require targeted policy interventions in Brazil.

The analysis of psychological wellbeing indicated generally high levels across all three economies, with Malaysia again recording the highest scores. Since teacher wellbeing encompasses multifaceted internal and external dimensions (Kern et al., 2014), these positive findings are encouraging for workforce sustainability. High wellbeing is consistently associated with better mental health for teachers and improved motivation for students (Hussain et al., 2022; Klusmann et al., 2021), suggesting that despite other systemic differences, teachers in these economies maintain a resilient psychological state.

Perceptions of principal leadership also exhibited economy-specific variations. Malaysian teachers perceived the most frequent leadership behaviors, whereas Macao teachers reported the lowest frequency, despite their system’s academic success. This finding aligns with observations that characterize teachers in certain East Asian systems as autonomous professionals or “generals” (OECD, 2014), a context in which overt instructional leadership might be less visible or enacted differently. These results underscore that leadership is not a monolithic construct but is shaped by the interaction between administrative practices and the broader policy environment of each economy.

### Implications of mediation analyses

Positive significant relationships were found between principal leadership, teachers’ wellbeing, job satisfaction, teacher autonomy, and organizational trust. Principal leadership was positively associated with wellbeing and job satisfaction. This finding supports the positive effects of principal leadership on teacher wellbeing, job satisfaction, professional practices, and organizational commitment in previous studies (e.g., Bellibaş et al., 2020; Crisci et al., 2019; Halim et al., 2021; Martinsone et al., 2024; Nguni et al., 2006; Qi et al., 2025; Zhong et al., 2020). Beyond these linear associations, a key finding of this study is the mediating role of teacher autonomy and organisational trust. Both factors significantly mediated the relationships between principal leadership and job satisfaction, and between leadership and wellbeing. These results demonstrate that, while principal leadership is positively associated with teachers’ job satisfaction and wellbeing, its influence is primarily exerted through indirect pathways, granting teachers greater autonomy and cultivating a trust-based environment within the school. This underscores the importance of autonomy for job satisfaction, wellbeing, and productivity (Keddie et al., 2024), and suggests that principal leadership should actively support teacher autonomy (Worth and den Brande, 2020). An autonomous working environment is crucial for teachers to enjoy their profession (Skaalvik and Skaalvik, 2014) and for enhancing their job satisfaction and organizational commitment (Karataş and Güleş, 2010). Furthermore, creating a work culture based on trust is a fundamental leadership behavior that enhances organizational commitment (Cansoy and Polatcan, 2019). Therefore, school leaders’ encouragement of autonomy and fostering a culture of trust play a central role in translating leadership practices into improved teacher job satisfaction and wellbeing. Existing literature indicates that diverse leadership styles affect teacher outcomes differently. For example, transformational, laissez-faire, and servant leadership styles are often linked to high job satisfaction and low emotional exhaustion (e.g., Basar et al., 2021; Juhji et al., 2022; Saadaoui et al., 2024), whereas passive-avoidant and autocratic styles tend to have negative impacts (e.g., Halim et al., 2021; Rexha and Buleshkaj, 2024). The findings of the current study contribute to this body of work by showing that, regardless of the specific style, the perceived quality of leadership significantly influences teachers’ professional lives, predominantly by shaping the organizational conditions, specifically trust and autonomy, under which teachers work. Across the three economies, the mediation structure shows both common and nuanced economy-level patterns. Organizational trust and autonomy acted as robust mediators of the leadership–wellbeing relationship in all three systems. In contrast, job satisfaction mediated this relationship in Macao and Malaysia but not in Brazil. This distinct pattern in Brazil, in which job satisfaction serves as a weaker mediator, may be attributable to broader structural challenges. Literature highlights that Brazilian teachers often face precarious working conditions, including lower salaries than the OECD average and infrastructure limitations (OECD, 2023). In such a context, even when teachers are satisfied with their specific job roles, these overriding systemic and financial stressors may sever the link between job satisfaction and their overall psychological wellbeing. These results suggest that broadly similar mechanisms link leadership and wellbeing across education systems, even though the strength of individual pathways varies across economies. Because the study does not include direct indicators of labor-market conditions, accountability regimes, or cultural values, these between-economy differences should be regarded as tentative system-level patterns rather than firm causal explanations. Future comparative research that combines PISA-type survey data with macro-level indicators could more directly test such system-level hypotheses.

### Implications for leadership practice

The findings also translate into several concrete implications for school leadership practice across the three economies. First, the strong mediating role of organisational trust suggests that principals should consciously build fair and transparent school climates. This can include regular, open communication about decisions, consistent enforcement of school rules, and structured opportunities for teachers to voice concerns and contribute to school-wide discussions. Such practices are likely to reinforce teachers’ perceptions of institutional trust, which in turn supports both job satisfaction and psychological wellbeing.

Second, the mediating effect of professional autonomy indicates that school leaders should provide teachers with meaningful discretion over their teaching methods, classroom organisation, and aspects of assessment, while still aligning with curriculum and accountability expectations. Concrete strategies include allocating time for collaborative lesson planning, encouraging teacher-led innovation projects, and framing classroom observations as opportunities for professional dialog rather than primarily evaluative exercises. These practices can help teachers experience autonomy as supported rather than isolated, thereby strengthening the positive association between leadership and wellbeing.

Third, although job satisfaction did not mediate the leadership–wellbeing relationship in Brazil, the strong overall associations observed across economies underline the importance of monitoring and supporting teachers’ satisfaction. Principals can regularly check in with staff about workload, recognition, and professional growth opportunities, and they can advocate for realistic expectations regarding administrative duties and teaching loads. At the system level, policies that protect time for instructional leadership and teacher collaboration may amplify school-level efforts in all three economies, especially in high-stakes accountability contexts.

### Limitations and directions for future research

This study has several limitations that should be considered when interpreting the results. First, the research relies on cross-sectional data from PISA 2022. While the proposed serial mediation model specifies directional paths based on established theoretical frameworks, the cross-sectional nature of the data precludes the determination of definitive causal relationships. Therefore, the findings represent associations rather than confirmed causality, and future research employing longitudinal designs is recommended to test these causal mechanisms rigorously.

Second, regarding the inclusion of covariates, this study prioritized model parsimony to examine the structural invariance of the core theoretical constructs across diverse cultural contexts. Consequently, demographic covariates were not included as control variables in the structural model; instead, PISA sampling weights were applied in descriptive statistics to adjust for demographic distributions and ensure the findings are representative of the teacher populations in Brazil, Macao, and Malaysia.

Third, although the study compares three distinct economies, the dataset does not include direct measures of specific cultural values, labor market conditions, or accountability regimes. Consequently, the observed differences between Brazil, Macao, and Malaysia are interpreted as system-level patterns rather than effects of measured cultural variables. Future studies incorporating macro-level indicators could provide more granular insights into these cross-cultural variations.

Fourth, the data rely on self-reported measures from teachers, which may be subject to social desirability or response consistency biases inherent in survey research. Future research could benefit from integrating qualitative data or multiple informant perspectives to triangulate these findings.

Finally, a methodological limitation of the PISA dataset’s complex survey design should be noted. In the present study, sampling weights and adjustments for the clustering of teachers within schools were not applied in the structural equation modeling analyses. The primary focus of this research was to examine the invariance of structural relationships and theoretical associations across economies, rather than to derive precise point estimates for population parameters. Consequently, the reported standard errors may be underestimated, increasing the risk of Type I errors. Therefore, the results should be interpreted as findings reflecting the characteristics of the analyzed sample rather than fully design-adjusted population estimates. Future research utilizing multilevel modeling techniques or robust standard error adjustments is recommended to validate these structural pathways further while explicitly accounting for the nested nature of the data.

## Conclusion

This study revealed differences across economies in teachers’ perceptions of principal leadership, wellbeing, job satisfaction, professional autonomy, and organisational trust in Macao, Malaysia, and Brazil. One notable result of the study is that principal leadership influences teachers’ job satisfaction and wellbeing predominantly through indirect mechanisms, mediated by factors such as professional autonomy and organizational trust. The study emphasises the importance of positive school climate and supportive leadership approaches in teachers’ professional lives. School leaders’ support for autonomy and for creating a trusting environment is crucial to enhancing teachers’ job satisfaction and wellbeing. Taken together, the broadly similar mediation patterns observed across these three economies suggest that strengthening leadership, autonomy, and organizational trust may be a promising strategy for supporting teacher wellbeing in diverse education systems, even though the exact configuration of these resources differs across economies.

## Data Availability

The raw data supporting the conclusions of this article will be made available by the authors, without undue reservation.

## Footnotes

1
https://www.oecd.org/en/data/datasets/pisa-2022-database.html
